# Enriching cross-sectoral collaboration to foster inclusive cultures in schools: a Model to address the needs of diverse Chilean Students

**DOI:** 10.3389/fpsyg.2024.1356642

**Published:** 2024-06-20

**Authors:** Veronica Vidal, Susana Mendive, Florencia Gómez Zaccarelli, Francisca Pozo-Tapia, Marigen Narea, Daniela Wachholtz, Carolina Melo

**Affiliations:** ^1^Escuela de Fonoaudiología, Universidad de los Andes, Santiago, Chile; ^2^Departamento de Epidemiología y Estudios en Salud, Universidad de los Andes, Santiago, Chile; ^3^Facultad de Educación, Pontificia Universidad Católica de Chile, Santiago, Chile; ^4^Center for the Study of Policies and Practices in Education (CEPPE UC), Santiago, Chile; ^5^Departamento de Fonoaudiología, Pontificia Universidad Católica de Chile, Santiago, Chile; ^6^Escuela de Psicología, Pontificia Universidad Católica de Chile, Santiago, Chile; ^7^Centro de Estudios Avanzados en Justicia Educacional (CJE), Santiago, Chile; ^8^Escuela de Terapia Ocupacional, Universidad de los Andes, Santiago, Chile; ^9^School of Education, Universidad de los Andes, Santiago, Chile

**Keywords:** inclusive education, Chile, cross-sectoral collaboration, school, diverse students

## Abstract

Inclusive education involves the interaction of diverse actors from different societal sectors, such as education, health, and policy. Inclusion laws and regulations in Chile are relatively new and have been taken as a regional model. However, the efforts to implement them have revealed some structural difficulties that must be discussed. This conceptual analysis article aims to provide insights to enrich cross-sectoral collaboration to foster inclusive cultures in Chilean schools. Considering the OECD Analytical Framework, which describes a systemic approach, we provide definitions for the critical components of the model and discuss the advances and challenges of current Chilean public policies in this field –including the Chile Crece Contigo and the School Integration Programs (SIP)—, the Chilean education system functioning, the social contexts, and students’ needs and supports based on the available evidence. Building from inclusive education literature and previous experiences, we delve into the model to address the needs of students with disabilities, social and cultural disadvantages, students belonging to the indigenous population, and students with a low socioeconomic level to propose action guidelines with a particular focus on integrating inclusive practices at the school level.

## Introduction

Inclusive education is broadly recognized as a right for all and an essential challenge to the educational system at different levels (UNESCO, 2016). It is a process that fosters overcoming the barriers that limit the presence, participation, and performance of all learners in educational institutions (UNESCO, 2016). Inclusive education is about changing the system to fit the student, not changing the student to fit the system (Farkas, 2014). Inclusive education involves understanding that intersectionality –interconnected social categorizations, such as race, socioeconomic status, gender, and disability– determines students’ educational needs and accommodations requirements (Bešić, 2020). When intersectionality is not addressed, lines of action intended for inclusion may lead to discriminatory processes (Bešić, 2020). Inclusive education also requires cross-sectoral efforts, namely integrative and collaborative work between different societal sectors, including but not limited to education, health, and social services, to articulate policy and practice efforts and supply appropriate responses to the educational needs of diverse students (Veríssimo de Farias et al., 2016; Patana, 2020). Providing inclusive education means integrating policies with practices to employ economic, human, and material resources to offer an educative response to students, considering their particular needs (Patana, 2020).

Aligned with human rights conventions and international laws and UNICEF’s Public Finance Strategy for Children (United Nations, 2006; OCDE, 2012; De Beco et al., 2019; Art.24; World Bank, 2011; UNICEF, 2017), Chile has developed a set of laws and regulations to promote inclusive education based on its regulatory foundations (i.e., Ministry of Social Development and Family, 1993; Ministry of Planning and Cooperation, 1993; Ministry of Education, 2008; Ministry of Education, 2009a; Ministry of education, 2009b; Ministry of Education (2015a); Law 20.845 of MINEDUC, 2015a; Ministry of Social Development and Family, 2022; Ministry of Health, 2023; Ministry of Education, 2023). Chile Crece Contigo—and the School Integration Program (SIP)—also called PIE for its acronym in Spanish—have been a breakthrough for inclusive education. Still, they face critical challenges such as financial resources and limited cross-sectoral collaboration (e.g., Milman et al., 2018; Tamayo-Rozas et al., 2018; Patana, 2020).

### Chilean inclusive education policy framework

In Chile, some fundamental laws and systems open the space for inclusive education, such as the *Law of Guarantees and the comprehensive protection of the rights of children and adolescents* (Law 21.430 of 2022), which promotes the creation of a multi-sector and multi-level Guarantee System (i.e., legislative, judicial, state agencies and local governments). This law has started its implementation gradually since 2022. It guarantees the rights of children and adolescents, including “the right to be educated in the development of their personality, aptitudes, and capacity development” [Law 21.430 of 2022, article 41; see also Defensoría de la Niñez, 2023]. In addition, this law guarantees the right to receive education beyond any condition of children’s lives and access to regular education for disabled children (Law 21.430 of 2022). One year after its implementation, several warnings were raised by an autonomous organism called Defensoría de la Niñez (Children’s Ombudsman), which advocates for the rights of children and adolescents living in Chile. Among them is the need to implement an inter-institutional board to ensure cross-sectoral work, clarifying the roles of each stakeholder (i.e., health and Education) (Defensoría de la Niñez, 2023). Effective cross-sectoral work is vital for the success of the Guarantee System, and the warning seems reasonable, considering the implementation of a previous cross-sectoral effort called Chile Grows with You (Chile Crece Contigo, ChCC) [e.g., González-Burboa et al. (2020) and Rossi and Parada (2023)]. ChCC is a comprehensive protection system for children from prenatal to 9 years old,^1^ which provides health and coordinated services across different public sectors to support child development (Peralta, 2011; Arcos-Griffiths et al., 2016; Torres et al., 2018). One of the critical challenges of ChCC is to adapt the range and type of services provided, such as the management of children with disabilities or new immigrants and indigenous populations (Milman et al., 2018; Grau-Rengifo et al., 2022).

Specifically for laws that regulate the Chilean educational system, the first efforts to promote inclusive education were born with Decree 490 to integrate students with special educational needs into general education classrooms, the law of intercultural education (Law 19.253 of 1993), and the law for the full inclusion of disabled individuals (Law 19.284 of 1993). In 2000, a set of regulations emerged on this topic. In particular, the law of Preferential School Subsidy (known as *SEP* law) was declared (Law 20.248 of 2008) to improve the quality of schools that enroll students from low socioeconomic backgrounds.

In addition, the General Education Law (Law 20,370 of 2009) regulates the rights and duties in the Chilean educational system. This law establishes education as a right of all people and promotes inclusion in the educational context of populations considered disadvantaged by gender, ethnicity, or socioeconomic status (Law 20,370 of 2009). Even when the law mentions different minority groups, it defines special education as a specific transversal modality of the educational system [e.g., Secretaría de Educación Parvularia (2019). This modality provides services, resources, and support from professionals with specialized knowledge to students with temporary or permanent special educational needs (SEN), which can affect their development or access to learning (Sandoval et al., 2021).

An aspect of Chilean regulations is that special needs are officially recognized based on the health professional diagnosis, according to procedures described in the Ministry of education (2009b) that regulates access and support provided through the SIP. Focused on disabled students, a Decree for diversification of teaching passed in 2015 (Decree 83 of 2015), prescribing the Universal Learning Design (Rose, 2000; Rose and Meyer, 2000) for classroom teaching in schools at the national level. During the same year, the senate passed the school inclusion law [Ministry of Education (2015b)] to regulate school access and profit. Law 20.845 states that the system should respect and foster diversity in its broad spectrum in schools, including students from different cultures, religions, socioeconomic status, and special education needs. In addition, the miscellaneous law [Ministry of Education (2023)] ensures 5% of private school vacancies for students with permanent SEN.

Even though Chile’s regulatory framework shows significant progress toward the goal of inclusion, a series of studies on the implementation of SIP in Chile identify tension and ambivalence between regulations and pedagogical practices (López et al., 2014; Urbina Hurtado et al., 2017; Tamayo-Rozas et al., 2018; Arriagada Hernández et al., 2021; Sandoval et al., 2021). In this regard, there are two critical issues: (1) The incentive mechanism for diverse students’ inclusion through the funding of students by voucher (Varela et al., 2015; Bas, 2021), and (2) the lack of cross-sectoral collaboration (Patana, 2020). We focus on discussing the lack of cross-sectoral collaboration from a conceptual perspective. It addresses more immediate solutions to local communities and constitutes a less structural change, but has the potential to foster systemic changes [e.g., Dimmock et al., 2021; Sahlberg, 2021)].

For this conceptual article, we use Cerna et al.’s (2021) framework to analyze the inclusive education policies implemented in the Chilean context. Applied from what is proposed by Beaney (2010) and Bechtel and Richardson (2010), we employed conceptual analysis techniques primarily to break down the key components or constituent parts of the analytical framework (Cerna et al., 2021) to gain knowledge of the design aspects of the policy and offer insights for future policy situated in a specific context, in this case, Chile.

Based on a resilience framework (OECD, 2019) and with the influence of Bronfenbrenner’s ecological model of human development (Bronfenbrenner and Morris, 2007), Cerna et al.’s (2021) framework offers a systemic approach to assess whether societies are considering the different factors and actors to develop an educational response to the intersectional nature of diversity. As a result, we aim to identify challenges and provide insights on enriching cross-sectoral collaboration to foster inclusive cultures in Chilean schools.

## Challenges for inclusive education becoming a reality in Chile

**Figure 1:**
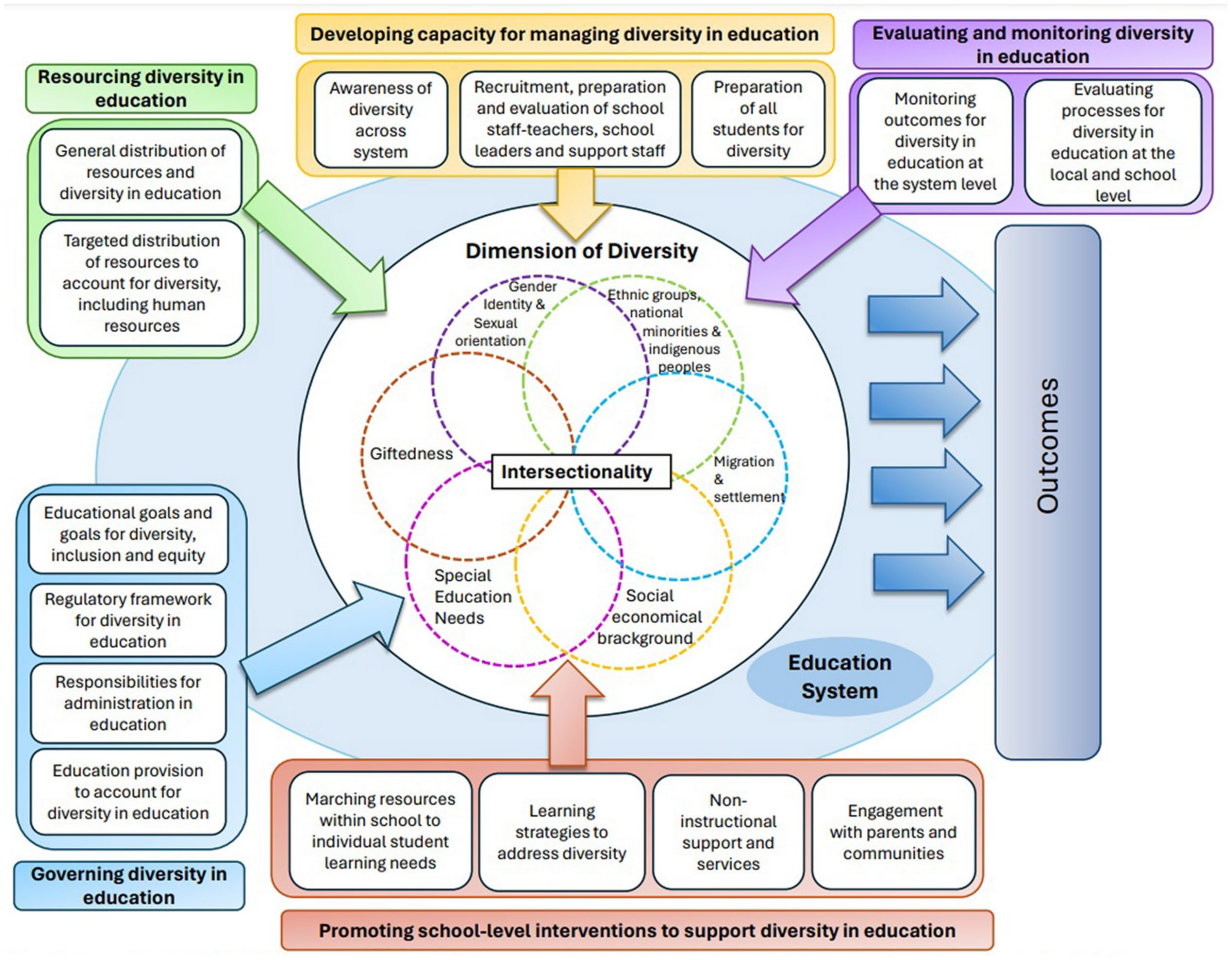
Cerna et al. (2021) Analytical framework for inclusive education. Analytical framework for developing inclusive education systems responsive to diverse population from “Promoting inclusive education for diverse societies: A conceptual framework,” by Cerna et al. (2021), in OECD Education Working Papers, No. 260, OECD Publishing, Paris, https://doi.org/10.1787/94ab68c6-en.

Cerna et al.’s (2021) framework claims that inclusion involves more than the student and organizes the factors in a multilevel approach, including microsystem actors, such as students and their families and teachers and support staff; mesosystem actors, such as school leaders; exosystem actors, such as the educational system, and macrosystem actors, such as society and their attitudes (Cerna et al., 2021). Based on identifying these actors, the framework suggests five key issues that we will treat as concepts to conduct this conceptual analysis: (a) governance, (b) resourcing, (c) capacity development, (d) school-level interventions, (e) monitoring outcomes, and evaluating processes for diversity in education (Cerna et al., 2021); all contained in a societal system that includes the social context, the policy, and the legal frameworks (see Figure 1). Accordingly, in the following paragraphs, we describe the challenges that the Chilean educational system faces, organized by the five critical issues proposed by Cerna et al.’s (2021) analytical framework.

### Governance: institutional challenges

#### Definition and implementation in Chile

According to Cerna et al. (2021), governance includes educational goals for diversity, inclusion, and equity, as well as the regulatory framework and responsibilities for and administration of diversity to provide diversified education. Particularly in Chile, education is structured through a national curriculum. The highest authority in education is the Ministry of Education, which promulgates the national curriculum, organized into mandatory minimum objectives and contents that act as expected standards for school learning at all levels.

#### Identified challenges

Law 20,845 establishes that the Undersecretary of Education, General Education Division, and in particular, the Education for All Unit, is responsible for providing guidelines to the educational system to promote the construction of inclusive educational communities (MINEDUC, 2020). However, several ministerial units issue guidelines linked to educational inclusion (e.g., inclusion of immigrants and belonging to indigenous peoples, coexistence program, citizenship policy, among others). For this reason, no single easily recognizable entity concentrates on delivering and evaluating the implementation of the guidelines to schools, resulting in high variability in the implementation and effectiveness of those guidelines across schools.

Cerna et al. (2021) recognizes the relevance of cross-sectoral collaboration in terms of responsibilities to ensure and provide inclusive education. Cross-sectoral responsibility needs to be implemented and monitored in Chile (Bedregal et al., 2014). It must be articulated at the national (i.e., Ministry of Education, Ministry of Health, Ministry of Social Development and Family, and the National Service of Migration) and local levels (i.e., regions, districts, and municipalities) (UNESCO, 2005; Rieser, 2012). Even when the Law of Guarantees and the System of Comprehensive Protection of the Rights of Children and Adolescents are considered advances in this issue, the education sector does not have actual participation (Defensoría de la Niñez, 2023).

Regarding education provision, Chile needs to work on systems to provide curricular adjustments to satisfy the particular needs of specific student populations. For example, the identity and language of the indigenous population are included only in schools with 20% of students from native cultures (Ministry of Education, 2017a; Gutiérrez-Pezo, 2020). This reality limits the opportunity to satisfy the fundamental right of identity of indigenous students who do not attend these schools (Poblete, 2019).

### Resourcing

#### Definition and implementation in Chile

According to Cerna et al. (2021), resourcing for inclusive education involves providing funding and human capital to develop educational responses to diversity. Particularly for students with special educational needs (SEN), Decree 170 (MINEDUC, 2009; Ministry of Education, 2017b) allows support for these students in school contexts. This coverage has been provided through the SIP implemented in each enrolled school (Decree 170 of 2009). In Chile, there are three kinds of school administration: Private schools,^2^ subsidized schools –i.e., private schools that receive subsidies from the government–and public schools administered by municipalities (Mizala et al., 2002). Subsidies for inclusive education are provided only to schools that receive public resources. Private schools do not receive subsidies for inclusive education (Bas, 2021).

In addition to Decree 170, associated with student diversity beyond special education, the SEP law allocates an additional subsidy to students in vulnerable conditions (Law 20.248 of 2008). The resources are provided to each school’s administration, which commits to employing these resources in executing an education improvement plan for these students (Law 20.248 of 2008). According to the Ministry of Education’s website, based on 2023 data, 99% of public and 66% of subsidized schools receive this benefit.

#### Identified challenges

Even when positive changes have been documented after the promulgation of these two policies, several challenges have been identified. Decree 170’s implementation at schools is voluntary, and the decision of school teams to implement the SIP depends on the estimated resources that SIP would give to schools (Peña, 2013). These resources are subject to the number of students with a diagnosis, even when the voucher systems have been demonstrated to be an ineffective policy (McEwan and Carnoy, 2000). For example, small schools usually do not implement SIP because they need access to more vouchers to hire professionals to support children with SEN. This situation causes unequal conditions for those who study in differently sourced schools. It is even more evident in private schools, in which they select students according to characteristics that the schools desire from their potential students (Guerra-Araya, 2018). In addition to human capital, inclusive education involves allocating resources to count on adequate physical space or facilities (Benavides-Moreno et al., 2021). Just the willingness to create inclusive school programs and the available resources make the coverage of this policy insufficient to give an educative response to all students with SEN in all schools independent of their administrations.

Because of the voucher system associated with the implementation of Decree 170, the human capital available for the SEN programs is also limited. Teams of educational assistant professionals must concentrate their efforts only on children officially diagnosed with some temporary or permanent SEN because those children are considered to have subsidized support (Peña, 2013; San Martín et al., 2020). It restricts the number of professionals hired to implement inclusive education (Peña, 2013; San Martín et al., 2020). Then, the time available to support different aspects of diversity is also limited (Sandoval et al., 2021). Like they have scarce human resources, teachers and educational assistant professionals do not have enough time to collaborate (González-Burboa et al. (2020)) nor co-teach (Rosas and Palacios, 2021).

Conversely, evidence shows that SEP law has reduced the learning gap between schools with minors versus better socioeconomic conditions measured with the SIMCE test,^3^ but only at the middle levels (Treviño et al., 2023). Adverse consequences are reported on the autonomy and well-being of the teams due to the highly bureaucratic demand for accountability associated with allocating these resources (Treviño et al., 2023). In addition, the expenditure of resources related to this law is discretionary based on the priorities established by the school’s administrative manager. Even when part of these resources is oriented to hire professionals to support mainly socioemotional development, the work is isolated within an institutional policy with a lack of comprehension of these professionals’ approaches (López et al., 2011). Because of this, the remaining educational team has limited collaboration and support with these professionals (Gatica, 2015; Obando and Lamas, 2017). In sum, despite the financial efforts made by the Chilean regulations to support inclusive education practices at national and local levels, the voucher modality limits its underlying purpose of equity, and the system still lacks accurate knowledge on how to implement this policy to succeed.

### Capacity development

#### Definition and implementation in Chile

Schools must have aware communities and trained personnel to attend to the diversity in school settings appropriately (Cerna et al., 2021). In terms of awareness, an inclusive school and classroom requires a particular focus on the recognition and appraisal of the diversity of its students, both in the considerations for the achievement of learning goals as well as in the use of materials, methods, and assessment to achieve learning and participation (Sánchez-Gómez and López, 2020). Policies previously discussed were developed to favor the achievement of an inclusive classroom, providing better opportunities and scenarios to support vulnerable students during their school trajectories. At the school level, teachers have opportunities for training through the Center for Improvement, Experimentation, and Pedagogical Research of the Ministry of Education (CPEIP), which looks to enrich the knowledge and skills needed for professional practice (CPEIP, n.d.). Although the regulations mentioned above would favor this, the significant obstacle, again, is the implementation of inclusion at this level.

#### Identified awareness and cultural challenges

Besides the practical and political challenges of coordinating and implementing an inclusive education policy, there are often local challenges at the school level related to cultural beliefs and attitudes of families and teachers, which can sometimes be hard to change (Zoniou-Sideri and Vlachou, 2006; Hwang and Evans, 2011; Tiwari et al., 2015). Particularly in Latin American culture, there is a strong-rooted belief that children need hardship in education to develop strength and life skills and that equality for all is fair, just, and proper (Cheshire, 2019). These notions make inclusion hard for some community members, including secondary teachers, with a robust disciplinary background but fewer years of teaching and preparation for diverse students than early childhood or primary teachers (Boe et al., 2007; San Martin et al., 2021). Traditional Latin American beliefs of education can be particularly challenging when incorporating adaptations for students whose needs are not observable or evident to the bare eye (c.f., Morina, 2022). This may represent a solid invisible barrier that will make implementing an inclusion policy much harder (Morina, 2022).

In addition to the general belief that hard work in schools is a means to learning for life, specifically for students with SEN, school communities’ attitudes and beliefs usually align with a medicalized/deficit-based approach (Shakespeare, 2013; Vidal et al., 2023). Accordingly, schools focus on skills development rather than inclusion as education for all (Chieppa et al., 2023). Particularly for the Chilean case, Decree 170 perpetuates a medical and integrative model of support for educational needs by establishing clinical diagnostic criteria that orient the resources to support and maintain the stigma and segregation of these students (Sandoval et al., 2021; Zuñiga and Mansilla, 2023). In addition to students with SEN, available literature has documented gaps in cultural beliefs that negatively impact the requirements of other diverse groups such as LGTBQ and nonnative Spanish speakers’ students (Rojas Fabris et al., 2019; Jaramillo and Córdova, 2023). These areas call for enhanced awareness and targeted strategies to ensure inclusivity and effective education for all individuals, regardless of their background or identity.

*Identified challenges for teachers’ training.* Teachers in Chile have few tools and knowledge about inclusive education from their initial training and the formative support program offered by the CPEIP (Ministry of Education, 2016, 2017; OECD, 2019). The lack of specific training impacts the classroom because, even when teachers might believe in the relevance of inclusive education and be willing to apply strategies, teachers need to learn to use the most suitable approach to promote an inclusive environment appropriately (Crispel and Kasperski, 2021). An example of this issue is Universal Design for Learning (UDL). UDL implementation in teaching is mandated by decree, but not all teachers are trained to apply UDL principles in their teaching (Berríos Armijo et al., 2021). In addition, there is a need for a national entity that certifies the quality of in-service teacher professional development offerings that schools autonomously (Blanco, 2015). Therefore, teachers in its professional development and the whole educational teams require better appropriation of UDL (Berríos Armijo et al., 2021).

Another challenge is the knowledge needed to develop curriculum adjustments to address the needs of specific groups. For example, in Chile, offering education in indigenous languages, in addition to Spanish, is restricted to schools with 20% of children of a specific indigenous origin (Morales, 2021). Thus, the possibility of addressing the right of identity of indigenous children is not guaranteed for those who attend schools with a reduced percentage of this origin (Poblete, 2019).

In addition, Chilean classrooms are characterized as spaces where teaching practice is usually based on an Interrogation-Response-Evaluation type of interaction with limited student participation (Preiss, 2009; Radovic and Preiss, 2010). These rigid and sometimes authoritarian practices and physical barriers hinder inclusive classrooms (Zwane and Malale, 2018; Ireri et al., 2020). Accordingly, a change from a teacher-centered to a child-centered pedagogical perspective is needed.

### School interventions

#### Definition and implementation in Chile

At the school level, providing inclusive education requires making available the existing resources to meet the individual student’s needs. Current Chilean regulations limit the resources available to students. Resources oriented to assess and meet the needs of students with SEN at schools are described in Decree 170, which states the professionals and roles that these professionals play for each kind of diagnosed condition. Aligned with this reality, counting on cross-sectoral support outside the school is essential. To optimize the available resources and provide a comprehensive response to student needs, coordinated and articulate work between the school and primary health care services is relevant (Defensoría de la Niñez, 2023). Chile has developed policies to address cross-sectoral collaboration to support inclusive education, including Chile Crece Contigo, the Law of Guarantees, and the system of comprehensive protection of the rights of children and adolescents (Defensoría de la Niñez, 2023).

#### Identified challenges

Even when policies focusing on cross-collaboration toward inclusive education exist in Chile, in practice, there is a scarce collaboration between the school and primary health care services. Policies have been regulated and implemented independently by each sector. An example of a lack of cross-sectoral collaboration is how Decree 170 is implemented. In this case, the health sector participates in medical evaluations focused on diagnosis without the time and working conditions that encourage collaboration with teachers, which effectively may enhance learning (cf. Peña, 2013).

In addition, the theoretical and practical approaches that professionals working in these two contexts need to be better aligned (Bronstein, 2003; Peña, 2013; Duk et al., 2019). Health professionals working in a primary care service conceptualize students’ needs from a medicalized perspective, and they do not usually include intervention goals oriented to school readiness and performance (Torres et al., 2015). This clinical approach has clear disadvantages. Health professionals working with school-age children are more focused on treating the individual diagnosis than on essential skills to promote their learning. The objectives are decontextualized and unnatural, making it difficult to generalize these new skills in the pedagogical environment, everyday learning, and social and communicative situations. Besides, this model makes interprofessional collaboration unlikely (Smart and Smart, 2006). Thus, all of these characteristics generate segregation of children, leading to dissatisfaction in the support delivery based on this clinical model.

Another related issue is the integrality of the services provided at schools. According to de Decree 170, the diagnosis is essential to incorporate students into the SIP. However, only one diagnosis can be considered per child. The system does not allow an intersectional approach with support based on a student’s requirements or unrelated to their disability. For example, a student with a diagnosis of language disorder and socio-affective challenges due to the complexity of her family situation may only receive specialized support based on the language disorder diagnosis. This over-specificity restricts the hours of support and the kind of professionals this student can access weekly at schools. Therefore, opportunities for applying collaborative models with regular classroom teachers and holistic approaches focused on student well-being are scarce (Zuñiga and Mansilla, 2023).

### Monitoring

#### Definition and implementation in Chile

According to Cerna et al. (2021), monitoring of inclusive education should occur at different levels, including government authorities, school level, and student performance. This allows us to ensure the progress of the educational system toward becoming a real inclusive education. In Chile, systemic monitoring in schools is centralized in the Ministry of Education. Through the Regional Ministerial Secretariats (its acronym in Spanish: SEREMI), the Ministry of Education organizes and supervises schools in their jurisdictional territory, ensuring compliance with educational objectives and policies. At the school level, standardized monitoring systems have yet to be developed.

#### Identified challenges

Governmental monitoring at schools has focused on accountability rather than quality. An example of this practice is that subsidized schools are funded through a voucher system based on student attendance (López et al., 2018). This means the school administration receives funding according to the number of students attending. That funding is tied to access rather than the quality of education provided.

Similarly, policy mechanisms for inclusion in schools must be improved to guide practice or assess the quality of educational interventions for students participating in SIP (Zuñiga and Mancilla, 2023). Then, the school administration can use the funding to cover essential expenses, not necessarily those related to guaranteeing the quality of teaching or educational programs. In the case of private schools, since they do not receive public resources, monitoring is even less than in the other kind of schools. Therefore, more information is needed regarding how this portion of the educational system fosters inclusiveness and how effective it is. In such a scenario, developing more robust mechanisms to tie up resource allocation with procedures for its expenditure and quality monitoring to secure further funds is critical.

Another issue related to monitoring is the need to count with assessments focused on measuring the quality of inclusive education programs. This issue affects national and local-level decision-making due to insufficient evidence about the programs. Having bottom-up data developed at the school level offers possibilities for adapting policy to the needs of local contexts. In addition, this data provides information on the suitability of inclusive education programs to meet students’ needs appropriately (Chomba et al., 2014).

## Recommendations to enrich the cross-sectoral collaboration

Based on the identified challenges described in the previous section, we offer suggestions for the capacity development, school-level intervention, and the monitoring outcomes dimensions through the replacement of the PIE program by a universal multi-tiered system of support to foster the articulation between different sectoral actors in a shorter period with less macrosystemic changes (see also Bronfenbrenner and Morris, 2007).

A multi-tiered support system, or pyramid system of support, is an integrated and gradual system involving education and health professionals to respond to students’ educational needs. This system involves and seeks to efficiently connect financial professional resources and types of interventions according to the different levels of support needs (Burns et al., 2016). The pyramid system of support has been demonstrated to be successful in Finland, and it has been implemented since 2011 (Rosas et al., 2019). This system requires schools to build well-being and inclusion support teams. For this purpose, it is needed (a) a paradigm changes toward a well-being approach to education, (b) raising awareness, (c) building capacity for inclusive education, and (d) increasing the monitoring of inclusive education outcomes. A summary of suggestions is described in Table 1.

**Table 1:** Summary of Identified challenges and recommendations according to the critical issues proposed by Cerna et al. (2021).

OECD key issues	Identified challenges	Recommendations
Governance	Lack of implementation and monitoring of cross-sectoral responsibilities for inclusive education. Education Provision does not consider curricular adjustments for diverse groups.	
Resourcing	Funding is provided by each student. Expenditures are associated at the discretion of the management. Limited human resources to support a reduced number of students.	
Capacity development	Need for attitudes and perceptions changes of school communities about inclusive education. Limited training of teachers in inclusive education.	Raising awareness within school communities.
School-level interventions	Lack of consideration of students’ intersectionality. A gap in theoretical and practical approaches to conceptualize and support student needs.	Multi-tiered System of Support at the school level. Conformation of well-being teams.
Monitoring outcomes	Focus on measures of accountability instead of quality of education. Absence of assessment for inclusive education programs.	Increasing monitoring to the comprehensive learning diagnosis

*OECD, organization for economic co-operation and development.

### Paradigm change toward a well-being approach to inclusive education

In Nordic countries, the focus has changed from the student ‘issue’ to the student’s well-being (Hjörne and Säljö, 2004, 2014). Linguistically, it demonstrates a variation from a deficit-based language to a more positive one (Makoelle, 2020; Vidal et al., 2024). In terms of support, this change implies a turn of goals from eliminating or normalizing behaviors to providing strategies and accommodations for better quality-of-life outcomes. In practical terms, this change means a shift in focus. Meanwhile, the team still focuses on providing skills to reduce individual difficulties, and they are hired by a limited number of hours; the well-being team –as Nordic countries have visualized it— is part of the regular school staff and focuses on powering students (Hesjedal et al., 2016). In addition, these teams oversee coordinating cross-sectoral services to provide the necessary support to respond to the student’s needs (Hesjedal et al., 2016). See Mendive et al. (2024) for a detailed description of the suggested roles. This support organization would resolve the dispersion of educational and administrative guidance educational institutions receive on different issues related to inclusive education, such as children with SEN, social risk, coexistence plan, citizenship policy, and interculturality. To make sure that inclusive education meets the needs of all students under any condition, teams –working under the well-being approach– need to be coordinated by the school leadership team, which has to connect the guidelines to address inclusive education coming from the policy level with the vision and school’s language (Mendive et al., 2024).

### Raising awareness

An essential step in implementing inclusive education at the school level is sensitizing the community to inclusion and equity. It is necessary to provide a common language and educate the community on the importance of diversity and how the whole community and society ultimately benefit from inclusion.

Raising awareness requires identifying that students with diverse needs should be supported to become members of the educational community where they participate, even when they may be physically located in inclusive environments. Students who receive special services may need to be better integrated into the social ecology because the adults responsible for managing the room may need more ability to incorporate the interaction between academic, behavioral, communication, and social participation functioning domains (Carter, 2018; Farmer et al., 2019). A social ecology that supports students with diverse needs comes with an educational environment aware of students’ social roles and relationships. This dynamic contributes to teaching practices and behavior management in the classroom, designed for the educational community in an inclusive manner (Hamm et al., 2011). Then, it is relevant that school directors, teachers, and other members of the well-being team promote awareness of the social and support needs of students without concerning their abilities and their ethnicity, culture, or language (Carter, 2018) and ensure implementation, considering well-being teams and multi-tiered system of support.

### Building capacity for inclusive education

Regarding teacher professional development, we recommend incentivizing the whole school community to participate in educational inclusion training processes. According to Mendive et al. (2024), training should be organized at the school level, including monitoring inclusive educational practices. The goal is to build educational communities with diversity and inclusion as part of their culture (Duk et al., 2021).

### Increasing monitoring

Finally, a relevant condition for implementing a multi-tiered support system is to have valid evaluation instruments for the educational issues according to each level to collect regular and systematic data on student progress. Generally, we need to implement assessment measures oriented to quality instead of accountability. For example, developing a mechanism to ensure that the curriculum accommodates diverse students’ needs is necessary. In addition, methods of assessments that provide guidelines to ensure the quality of educational practices in inclusive education are highly needed (Zuñiga and Mansilla, 2023). In a multi-tiered system, monitoring methods that allow professionals to identify the level of support could be beneficial in providing better educative responses to students who have been frequently segregated in the traditional system.

Monitoring is also relevant to ensure a multi-tiered system’s implementation fidelity and assess students’ progress. Regarding fidelity, monitoring should be performed at the beginning and during the process. Initial monitoring is more successful than ongoing monitoring at the school level because planning is part of the mechanisms established in the school cultures and procedures. Therefore, schools should closely monitor the process (Nelson et al., 2015). For this purpose, we suggest that teachers take advantage of the Integral Learning Diagnostic Instrument (DIA by its acronym in Spanish). It is an instrument that allows measuring learning in language, math, and socioemotional areas at the individual and classroom level. This instrument could be an excellent resource to measure the outcomes of the pedagogical initiatives performed during the academic year. In addition, this instrument could also help teachers and the well-being team to identify the levels of support for their students (Mendive et al., 2024).

## Conclusion

This conceptual analysis paper sought to reflect on cross-sectoral collaboration efforts that can be made to foster inclusive education, taking Chile as an example. Using the OECD Framework for Inclusive Education (Cerna et al., 2021), we described the landscape of inclusive education in Chile and the cross-sectoral issues faced in making this approach to diverse students a real opportunity for inclusion. We know that a comprehensive and systemic commitment is needed to implement inclusive practices and policies successfully. Still, we state that local changes can foster a bottom-up change toward inclusive education that replaces a medicalized and fragmented view with a socio-cultural vision of education. The recommendations offered are oriented toward promoting the establishment of a well-being approach to inclusive education based on the successful experience of the Finnish model in implementing inclusion policies. The well-being approach comprehensively supports students and their intersectional needs through a collaborative and cross-sectoral effort. This approach requires heightened awareness at a social level to provide the grounds for inclusive perspectives in different sectors. It is also necessary to improve monitoring means to guarantee that all the laws and regulations already passed in the country can promote change and impact by providing inclusive education for all. We hope this conceptual analysis paper offers a view into one national reality and recommendations that serve as an image of the possibility for inclusive education in other countries.

## Author contributions

VV: Conceptualization, Writing – original draft, Writing – review & editing. SM: Conceptualization, Writing – original draft, Writing – review & editing. FG: Conceptualization, Writing – original draft, Writing – review & editing. FP-T: Conceptualization, Writing – original draft, Writing – review & editing. MN: Conceptualization, Writing – original draft, Writing – review & editing. DW: Conceptualization, Writing – original draft, Writing – review & editing. CM: Conceptualization, Writing – original draft, Writing – review & editing.

## Footnotes

^1^The program runs until the age of 9 because, according to the children’s advisory council, this is the period when the basis of different areas of development are built (Consejo Asesor de la infancia, 2006).

^2^Private schools only represent the 9% of the entire educational recruitment (Bravo et al., 2010).

^3^SIMCE is the National System for Measuring the Quality of Education, which employs census type of assessment in some subjects: language, math, science, and physical education.
